# Supplementary material for: Integrating AI/ML Models for Patient Stratification Leveraging Omics Dataset and Clinical Biomarkers from COVID-19 Patients: A Promising Approach to Personalized Medicine
Source: Int J Mol Sci. 2023 Mar 26;24(7):6250. doi: 10.3390/ijms24076250 (PMC10094615; doi:10.3390/ijms24076250)
Supplement: Supplementary file 1 [file ijms-24-06250-s001.zip › ijms-2284374-supplementary.pdf]

SUPPLEMENTARY DATA

Integrating AI/ML Models for Patient Stratification Leveraging Omics Dataset and Clinical Biomarkers from COVID-19 Patients: A Promising Approach to Personalized Medicine

Babatunde Bello, Yogesh N. Bundey, Roshan Bhave, Maksim Khotimchenko, Szczepan W. Baran, Kaushik Chakravarty and Jyotika Varshney

Table S1. Clinical data columns for ML workflow

Column Group	Column Group Definitions	Columns
Basic Profile	Basic profile data for patient	Age, Height (cm), Weight (kg), BMI
Comorbidities	Presence of external comorbidity	Alcoholic Nonalcoholic Liver Disease present, Asthma present, Atrial Fibrillation present, Cancer Flag present, Chronic Kidney Disease present, Chronic Viral Hepatitis present, COPD present, Coronary Artery Disease present, Crohn's Disease present, Diabetes present, Heart Failure present, Hypertension present, Obstructive Sleep Apnea present, Ulcerative Colitis present
Medications	Whether medication was given to patient	Apixaban given, Argatroban given, Azithromycin given, Dexamethasone given, Dobutamine given, Dopamine given, Eculizumab given, Enoxaparin given, Epinephrine given, Famotidine given, Gimisilumab given, Heparin given, Hydrocortisone given, Hydroxychloroquine given, Methotrexate given, Methylprednisolone given, Milrinone given, Norepinephrine Levophed given, No Ppm given, Phenylephrine given, Prednisone given, Remdesivir given, Rituximab given, Rivaroxaban given, Sarilumab given, Tissue Plasminogen Activator given, Tocilizumab given, Vasopressin Vasostrict given, Warfarin Coumadin given
Lab Results	Lab value of selected biomarker	Median Albumin lab value, Median Alt lab value, Median Anion Gap lab value, Median Ast lab value, Median Base Excess Arterial lab value, Median Base Excess Venous lab value, Median Basophil Number lab value, Median Basophil Percent lab value, Median Brain Natriuretic Protein lab value, Median Bun lab value, Median Calcium lab value, Median Carboxyhemoglobin Arterial lab value, Median Chloride lab value, Median Co2 Total lab value, Median Creatine Kinase Mb lab value, Median Creatine Kinase lab value, Median C Reactive Protein lab value, Median C Reative Protein Hs lab value, Median Deoxyhemoglobin Arterial lab value, Median D Dimer lab value, Median Egfr lab value, Median Eosinophil Number lab value, Median Eosinophil Percent lab value, Median Esr lab value, Median Ferritin lab value, Median Fibrinogen lab value, Median Glucose lab value, Median Haptoglobin lab value, Median Hco3 Arterial lab value, Median Hco3 Venous lab value, Median Hematocrit lab value, Median Hemoglobin A1C lab value, Median Hemoglobin Arterial lab value, Median Hemoglobin lab value, Median Inr lab value, Median Interleukin 1 Beta lab value, Median Interleukin 6 lab value, Median Interleukin 8 lab value, Median Lactate Arterial lab value, Median Ldh lab value, Median Lymphocyte Number lab value, Median Lymphocyte Percent lab value, Median Mchc lab value, Median Mch lab value, Median Mcv lab value, Median Mean Platelet Volume lab value, Median Methemoglobin Arterial lab value, Median Monocyte Number lab value, Median Monocyte Percent lab value, Median Neutrophil Number lab value, Median Neutrophil Percent lab value, Median O2 Saturation Arterial lab value, Median O2 Saturation Venous lab value, Median Oxyhemoglobin Arterial lab value, Median Pc02 Venous lab value, Median Pco2 Arterial lab value, Median Ph Arterial lab value, Median Ph Venous lab value, Median Platelet lab value, Median Po2 Arterial lab value, Median Po2 Venous lab value, Median Potassium lab value, Median Procalcitonin lab value, Median Prothrombin Time lab value, Median Ptt lab value, Median Rbc Count lab value, Median Serum Creatinine lab value, Median Sodium lab value, Median Tnf Alpha lab value, Median Total Bilirubin lab value, Median Total Protein lab value, Median Troponin I lab value, Median Uric Acid lab value, Median Wbc lab value, Maximum C Reactive Protein lab value, Maximum D Dimer lab value, Interquartile Range C Reactive Protein lab value, Interquartile Range D Dimer lab value, Minimum C Reactive Protein lab value, Minimum D Dimer lab value, Minimum Serum Creatinine lab value, Standard Deviation C Reactive Protein lab value, Standard Deviation D Dimer lab value, Mean C Reactive Protein lab value, Mean D Dimer lab value, Mean Mean Platelet Volume Median lab value, Number of C Reactive Protein observed - lab value, Number of D Dimer observed - lab value

Others	Maximum sequential organ failure assessment (SOFA) score recorded during the 24-hour period	SOFA Score

Table S2. Percentage of missing values per column within clinical biomarker training data

Column	Percent Missing (%)
Age	0
BMI	0.67
Alcoholic Nonalcoholic Liver Disease present	0
Asthma present	0
Atrial Fibrillation present	0
Cancer Flag present	0
Chronic Kidney Disease present	0
Chronic Viral Hepatitis present	0
Copd present	0
Coronary Artery Disease present	0
Crohns Disease present	0
Diabetes present	0
Heart Failure present	0
Htn present	0
Obstructive Sleep Apnea present	0
Ulcerative Colitis present	0
Height (cm)	2.5
Median Albumin lab value	23.99
Median Alt lab value	25.13
Median Anion Gap lab value	11.33
Median Ast lab value	25.6
Median Base Excess Arterial lab value	97.4
Median Base Excess Venous lab value	68.47

Median Basophil Number lab value	35.24
Median Basophil Percent lab value	20.09
Median Brain Natriuretic Protein lab value	91.86
Median Bun lab value	12
Median Calcium lab value	11.74
Median Carboxyhemoglobin Arterial lab value	83.5
Median Chloride lab value	10.87
Median Co2 Total lab value	11.72
Median Creatine Kinase Mb lab value	98.31
Median Creatine Kinase lab value	91.23
Interquartile Range C Reactive Protein lab value	39.96
Maximum C Reactive Protein lab value	39.96
Mean C Reactive Protein lab value	39.96
Median C Reactive Protein lab value	39.96
Minimum C Reactive Protein lab value	39.96
Number of C Reactive Protein observed - lab value	39.96
Standard Deviation C Reactive Protein lab value	94.68
Median C Reative Protein Hs lab value	98.7
Median Deoxyhemoglobin Arterial lab value	83.5
Interquartile Range D Dimer lab value	44.05
Maximum D Dimer lab value	44.05
Mean D Dimer lab value	44.05
Median D Dimer lab value	44.05
Minimum D Dimer lab value	44.05
Number of D Dimer observed - lab value	44.05
Standard Deviation D Dimer lab value	94.9
Median Egfr lab value	54.52
Median Eosinophil Number lab value	35.24
Median Eosinophil Percent lab value	20.09
Median Esr lab value	93.34
Median Ferritin lab value	42.01
Median Fibrinogen lab value	74

Median Glucose lab value	7.29
Median Haptoglobin lab value	97.87
Median Hco3 Arterial lab value	78.73
Median Hco3 Venous lab value	63.41
Median Hematocrit lab value	29.31
Median Hemoglobin A1C lab value	97.03
Median Hemoglobin Arterial lab value	80.91
Median Hemoglobin lab value	13.47
Median Inr lab value	47.76
Median Interleukin 1 Beta lab value	95.54
Median Interleukin 6 lab value	89.97
Median Interleukin 8 lab value	92.98
Median Lactate Arterial lab value	78.53
Median Ldh lab value	46.07
Median Lymphocyte Number lab value	35.24
Median Lymphocyte Percent lab value	20.06
Median Mchc lab value	13.49
Median Mch lab value	13.49
Median Mcv lab value	13.49
Mean Value lab value	13.87
Median Methemoglobin Arterial lab value	85.09
Median Monocyte Number lab value	35.24
Median Monocyte Percent lab value	20.06
Median Neutrophil Number lab value	18.93
Median Neutrophil Percent lab value	20.06
Median O2 Saturation Arterial lab value	78.86
Median O2 Saturation Venous lab value	62.93
Median Oxyhemoglobin Arterial lab value	79.69
Median Pc02 Venous lab value	60.26
Median Pco2 Arterial lab value	76.57
Median Ph Arterial lab value	76.59
Median Ph Venous lab value	60.27

Median Platelet lab value	14.58
Median Po2 Arterial lab value	76.58
Median Po2 Venous lab value	60.62
Median Potassium lab value	11.69
Median Procalcitonin lab value	58.49
Median Prothrombin Time lab value	47.76
Median Ptt lab value	48.68
Median Rbc Count lab value	13.49
Median Serum Creatinine lab value	11.79
Minimum Serum Creatinine lab value	11.79
Median Sodium lab value	25.47
Median Tnf Alpha lab value	92.98
Median Total Bilirubin lab value	24.28
Median Total Protein lab value	24.5
Median Troponin I lab value	87.67
Median Uric Acid lab value	95.5
Median Wbc lab value	13.7
Apixaban given	0
Argatroban given	0
Azithromycin given	0
Dexamethasone given	0
Dobutamine given	0
Dopamine given	0
Eculizumab given	0
Enoxaparin given	0
Epinephrine given	0
Famotidine given	0
Gimisilumab given	0
Heparin given	0
Hydrocortisone given	0
Hydroxychloroquine given	0
Methotrexate given	0

Methylprednisolone given	0
Milrinone given	0
Norepinephrine Levophed given	0
No Ppm given	0
Phenylephrine given	0
Prednisone given	0
Remdesivir given	0
Rituximab given	0
Rivaroxaban given	0
Sarilumab given	0
Tissue Plasminogen Activator given	0
Tocilizumab given	0
Vasopressin Vasostrict given	0
Warfarin Coumadin given	0
SOFA Score	2
Weight (kg)	1.75

Table S3. Clusters of patients with percent of severe patients per cluster and cluster biomarker observations. Clusters were generated through K-Means clustering where optimal number of clusters were determined through the Elbow Method. The data used as the input for clustering was SHAP values (feature impact scores) for the overall clinical feature set.

Cluster	Percent Severe (%)	Observations
**1**	89.3	- Patients with higher weight
**2**	10.9	- Patients with lower weight
**3**	94.6	- Patients with high SOFA score, BUN, and Serum Creatinine levels
**4**	7.3	- Patients with low SOFA score, BUN, and Serum Creatinine levels
**5**	93.1	- No real indicator<br>- Interesting group of patients
**6**	4.9	- Patients with high BUN, Serum Creatinine, LDH<br>- Low PCO2 arterial
**7**	6.2	- Patients with low values overall
**8**	93.4	- Patients with high LDH
**9**	92.8	- Patients with high potassium
**10**	1.7	- Patients with high lymphocyte percent
**11**	95.1	- Patients with high SOFA score, BUN, and Serum Creatinine levels
**12**	96.5	- Low BUN, Serum Creatinine<br>- High CO2 total
**13**	98.6	- High SOFA score, BUN<br>- Low Albumin<br>- Normal Serum Creatinine
**14**	11.1	- Low PCO2 Arterial<br>- Low SOFA score
**15**	96.0	- Normal SOFA score<br>- High BUN

Table S4. Common significant genes (at GS>0.3 & MM>0.8) in MEcyan module across traits - EOD, SOFA, BUN

CSL1	DYSF	LRRC4	RN7SL731P
ACTN1	EXOC6	MANSC1	RNF24
ADAM9	F5	MAP2K6	RNU7-45P
ADGRG3	FAM53C	MAPK14	ROPN1L
ALOX5	FCAR	MCTP2	RPL11P3
ALPK1	FGR	MEGF9	RPL26P5
ANO10	FLOT1	MGAM	RPS2P14
ANXA3	FLOT2	MIR3605	RTEL1P1
ATP11A	GCA	MIR4802	SEMA4A
ATXN1	GLT1D1	MIR7848	SIPA1L2
B4GALT5	HRH2	MKNK1	SIRPA
BASP1	HYCC2	MTCO3P23	SLC22A4
BASP1-AS1	IL17RA	MTCYBP23	SLC25A44
BAZ1A	ITGAM	MTMR3	SLC2A3
BCL6	JAK3	MTND3P12	SLC37A3
C3orf86P	KIF1B	MTND4LP23	SLC9A8
CAMKK2	KLHL2	MTND5P32	SLED1
CARD6	LILRA6	NABP1	SRPK1
CFLAR	LILRB3	NEAT1	SSH1
CR1	LRG1	NQO2	STAT5B
CRISPLD2	PIK3CD-AS1	PFKFB3	STX11
DRAM1	PLXNC1	PGS1	STX3
RALB	PPP1R3D	PHF21A	TGFA
RASGRP4	PRKCD	PHTF1	TLR5
RN7SL552P	PYGL	UPP1	TLR8
WDFY3	TRIM25	USB1	TMCO3
XPO6	UBXN2B	ZNF415P1	

Table S5. Common significant genes (at GS>0.2 & MM>0.8)in MEdarkred module across traits - EOD, SOFA, BUN, and heart failure, alcohol liver disease, kidney disease selected (at GS>0.1 & MM>0.8, since these set are only moderately significant)

ACSL1	LOT1	MTCYBP23	SEMA4A
ACTN1	FLOT2	MTMR3	SIPA1L2
ADAM9	GCA	MTND3P12	SIRPA
ADGRG3	GLT1D1	MTND4LP23	SLC22A4
ALOX5	HRH2	MTND5P32	SLC25A44
ALPK1	HYCC2	NABP1	SLC2A3
ANO10	IL17RA	NEAT1	SLC37A3
ANXA3	ITGAM	NQO2	SLC9A8
ATP11A	JAK3	FGR	SLED1
ATXN1	KIF1B	PFKFB3	SRPK1
B4GALT5	KLHL2	PGS1	SSH1
BASP1	LILRA6	PHF21A	STAT5B
BASP1-AS1	LILRB3	PHTF1	STX11
BAZ1A	LRG1	PIK3CD-AS1	STX3
BCL6	LRRC4	PLXNC1	TGFA
C3orf86P	MANSC1	PPP1R3D	TLR5
CAMKK2	MAP2K6	PRKCD	TLR8
CARD6	MAPK14	PYGL	TMCO3
CFLAR	MCTP2	RALB	TRIM25
CR1	MEGF9	RASGRP4	UBXN2B
CRISPLD2	MGAM	RN7SL552P	UPP1
DRAM1	MIR3605	RN7SL731P	USB1
DYSF	MIR4802	RNF24	WDFY3
EXOC6	MIR7848	RNU7-45P	XPO6
F5	MKNK1	ROPN1L	ZNF415P1
FAM53C	MTCO3P23	RPL11P3	RPS2P14
FCAR	TEL1P1	RPL26P5	

Table S6. Comparison of Balanced Accuracy and ROC-AUC among various modeling architectures for predicting COVID-19 severity. Boosted Decision Tree models, such as XGBoost, LightGBM, and CatBoost, all have built in methods for handling missing data, so evaluation metrics for these modeling architectures were reported with no data transformations and with MICE imputation. All other modeling architectures required missing data imputation, so MICE imputation was used.

	Balanced Accuracy	ROC-AUC
**LightGBM**	0.916	**0.981**
XGBoost	**0.925**	0.978
CatBoost	0.918	0.975
XGBoost + MICE	0.92	0.976
LightGBM + MICE	0.915	0.973
CatBoost + MICE	0.913	0.973
SVC + MICE	0.858	0.944
RandomForest + MICE	0.885	0.961
MLP + MICE	0.627	0.629

Table S7. Comparison of Balanced Accuracy and ROC-AUC among various modeling architectures for predicting COVID-19 survival. Boosted Decision Tree models, such as XGBoost, LightGBM, and CatBoost, all have built in methods for handling missing data, so evaluation metrics for these modeling architectures were reported with no data transformations and with MICE imputation. All other modeling architectures required missing data imputation, so MICE imputation was used.

	Balanced Accuracy	ROC-AUC
**LightGBM**	**0.995**	**0.999**
XGBoost	0.992	**0.999**
CatBoost	0.992	0.998
XGBoost + MICE	0.994	0.998
LightGBM + MICE	0.992	0.998
CatBoost + MICE	0.992	0.998
SVC + MICE	0.807	0.923
RandomForest + MICE	0.871	0.988
MLP + MICE	0.553	0.584

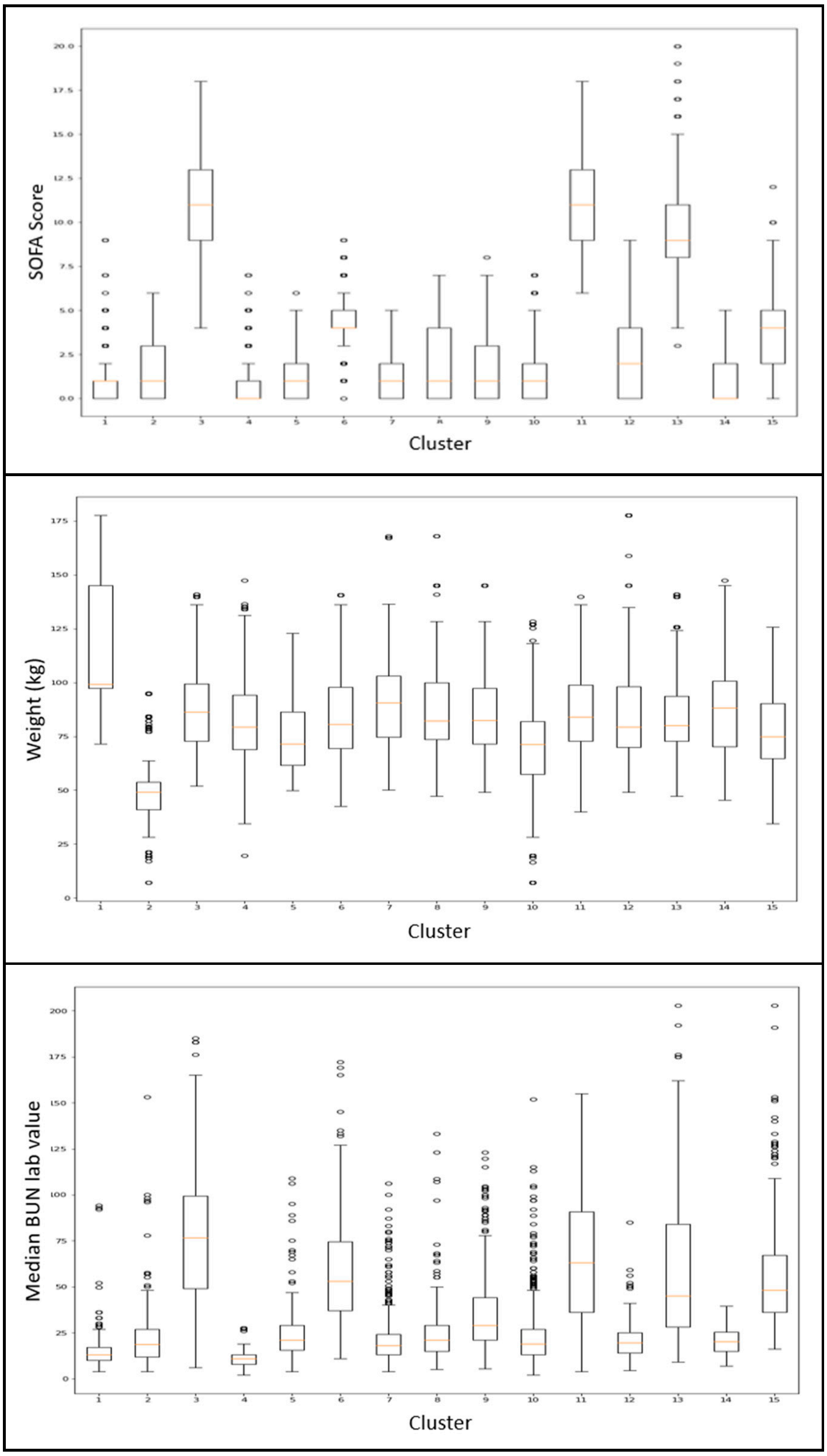
SOFA Score
Cluster
Weight (kg)
Cluster
Median BUN lab value
Cluster

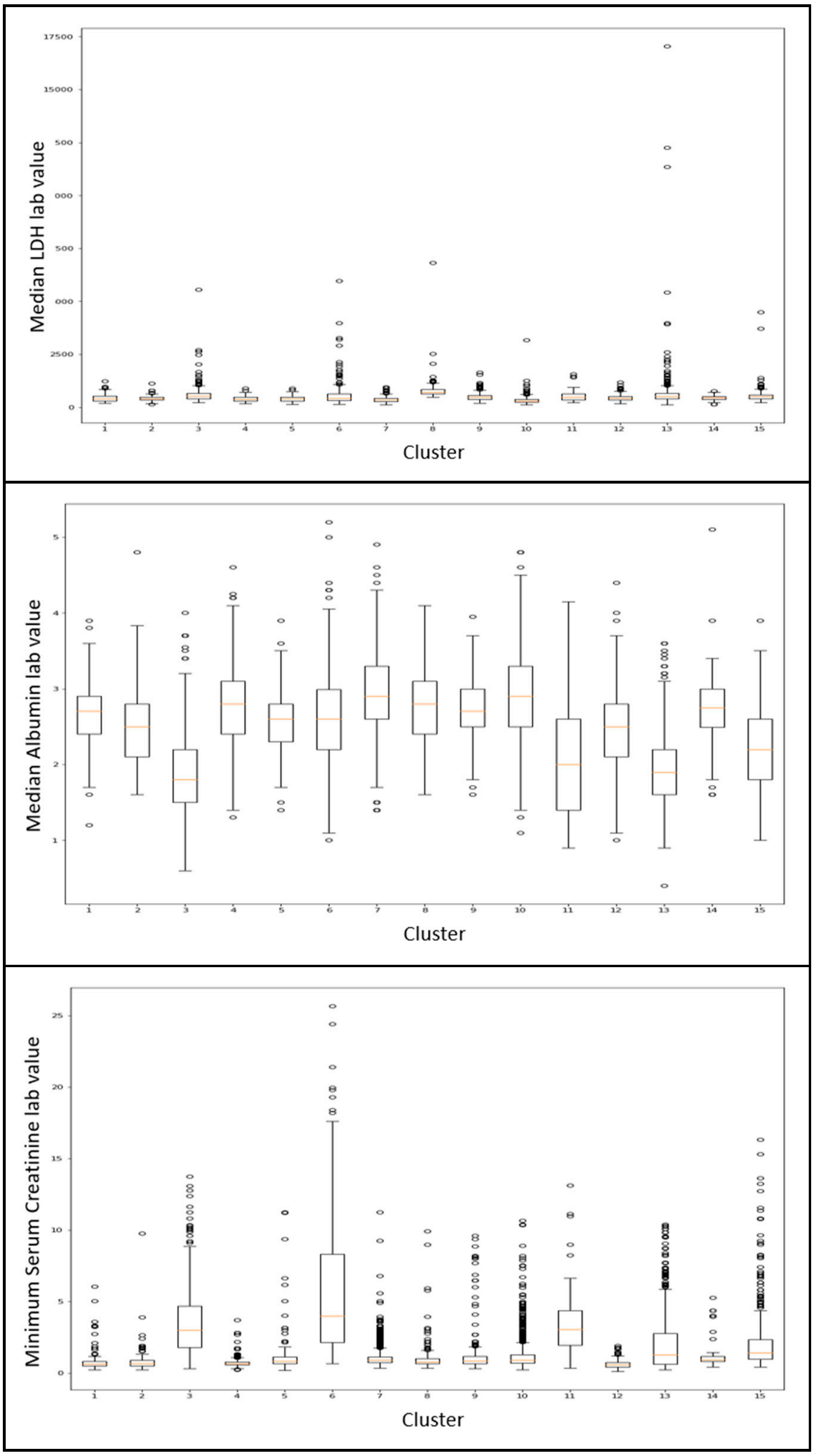
Median LDH lab value
17500
15000
500
000
500
000
2500
0
1 2 3 4 5 6 7 8 9 10 11 12 13 14 15
Cluster
Median Albumin lab value
5
4
3
2
1
1 2 3 4 5 6 7 8 9 10 11 12 13 14 15
Cluster
Minimum Serum Creatinine lab value
25
20
15
10
5
0
1 2 3 4 5 6 7 8 9 10 11 12 13 14 15
Cluster

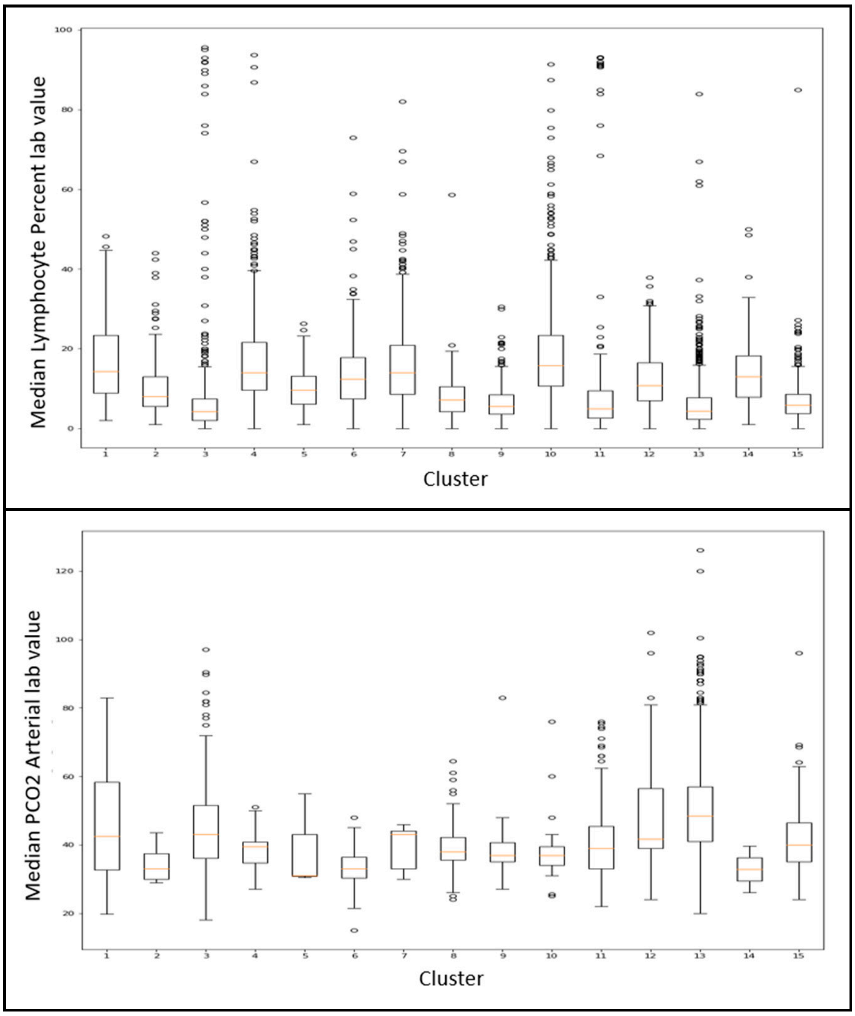

Figure S1. Biomarker value boxplots per cluster. Varying biomarker value thresholds can be clearly correlated with percentage of severe patients for many of the clusters. Cluster 5 did not have a clearly observed correlation between biomarker values and percent of severe patients. Much of the clusters that had high percent of severe patients had distinct levels of BUN, Serum Creatinine, and SOFA score.

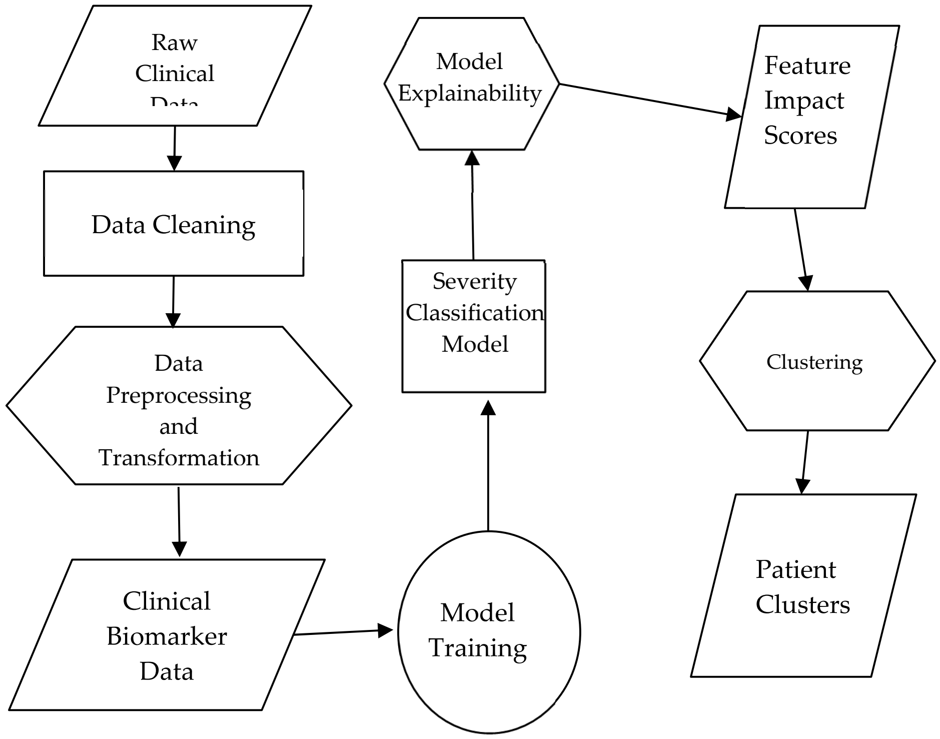

Figure S2. ML Clustering Methodology overview. Raw clinical data was cleaned and preprocessed to include relevant columns and appropriate value standardization. Clinical biomarker data was used in ML modeling for predicting COVID-19 case severity. Model explainability with SHAP was applied on the trained severity model and clinical biomarker data was transformed into feature impact scores, which was the input for clustering.

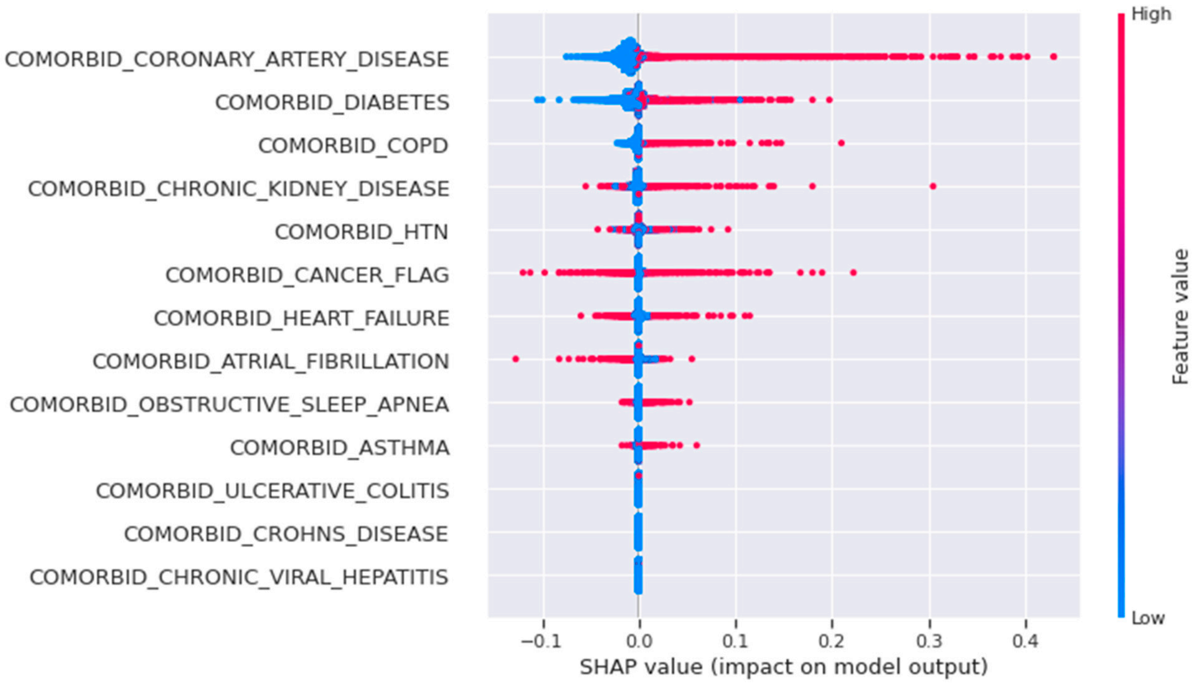

Figure S3. Comorbidity based inferences for outcome/survivability models seen via SHAP value beeswarm

Note

The bioinformatics analysis is to identify gene networks that show significant differences associated with COVID-19 severity, patient disease comorbidities, clinical biomarker measure during the patient stay in the clinic. The enrichment analysis is done on the significant gene network list using the GO (biological processes) and KEGG pathway for gene annotation.

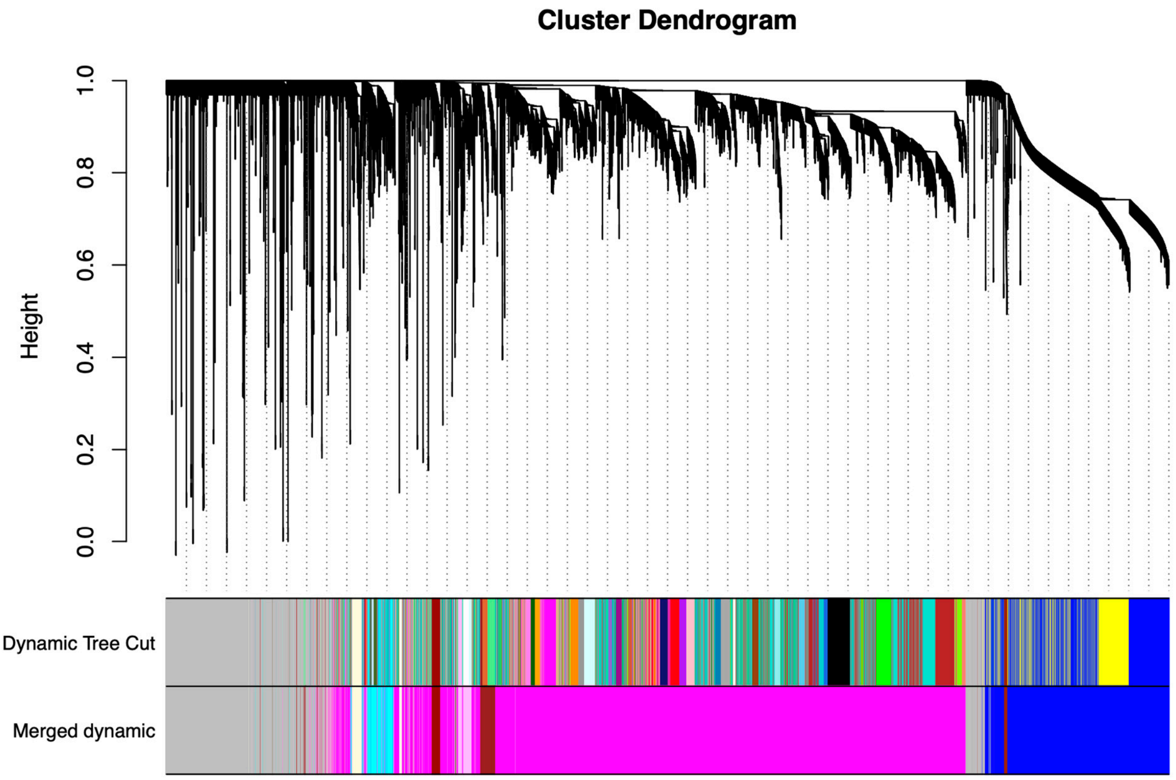

Figure S4 . Gene Cluster Dendrogram for. The blocks in Dynamic Tree Cuts are different modules or clusters of gene network, and Merged dynamic blocks modules merged together based on module similarity from Dynamic Tree Cut blocks

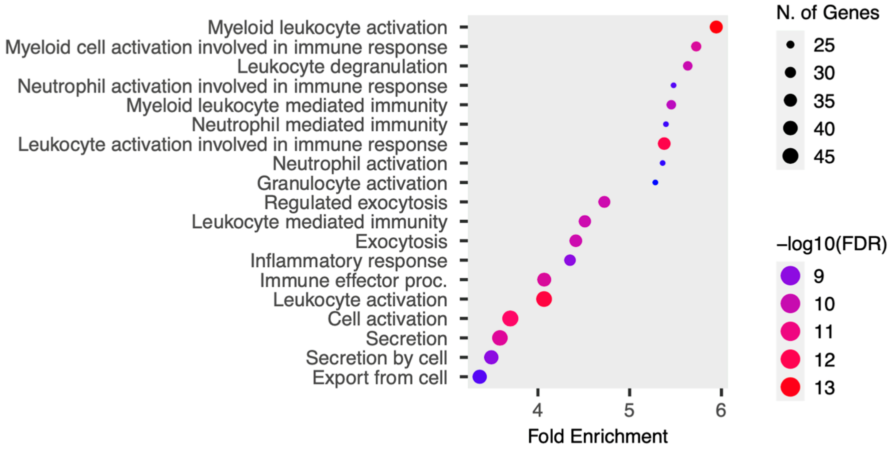

Figure S5. Dot plot for top 20 enriched pathways based for gene module, MEcyan based on GO annotation

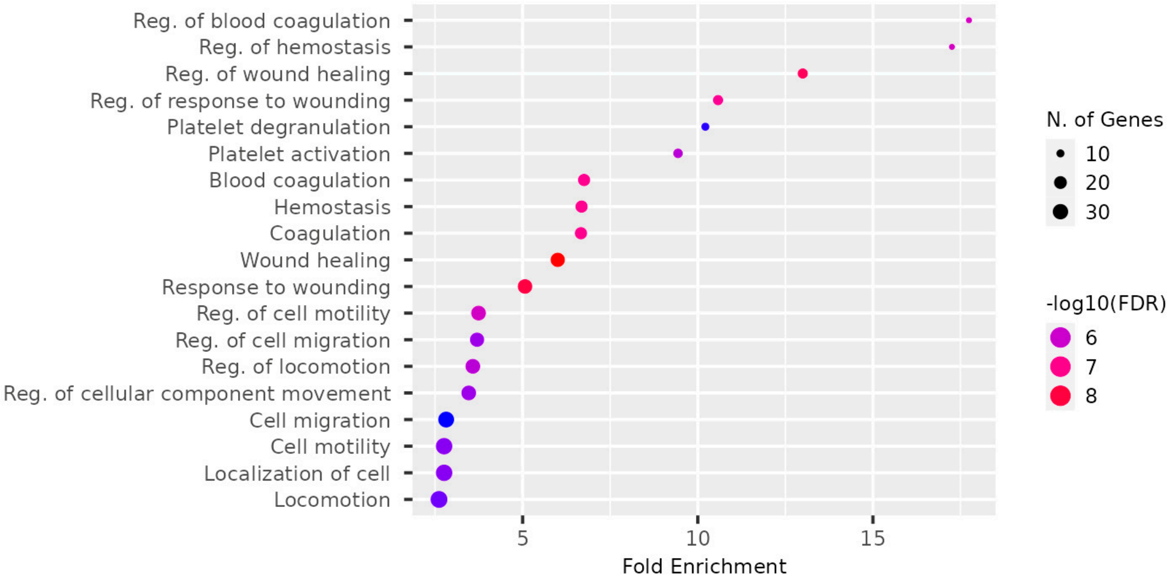

Figure S6. Dot plot for top 20 enriched pathways based for gene module, MEdarkred based on GO annotation

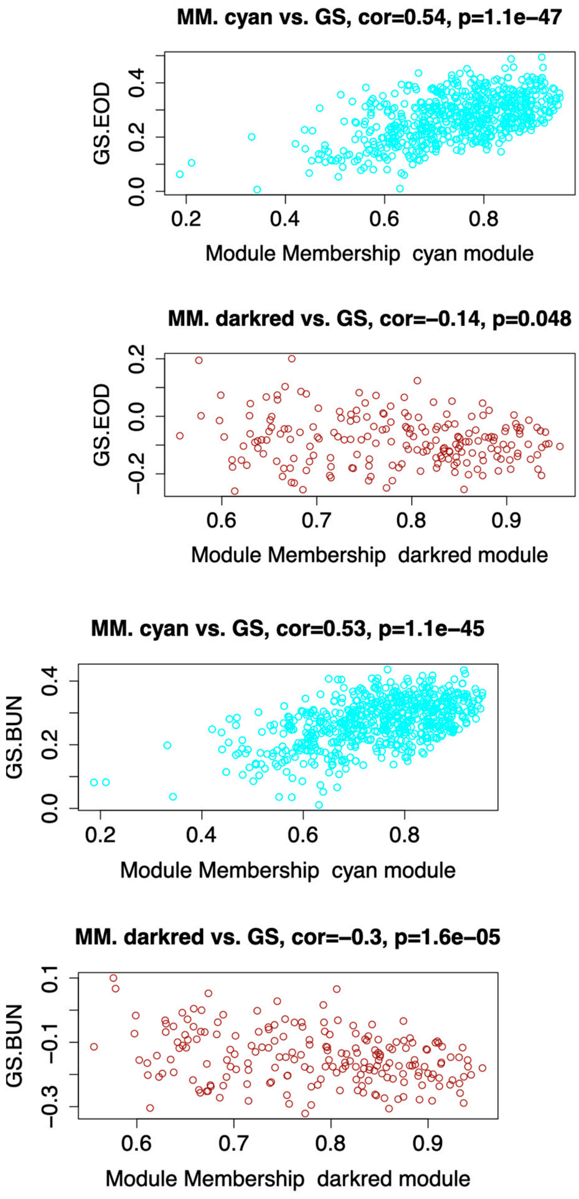

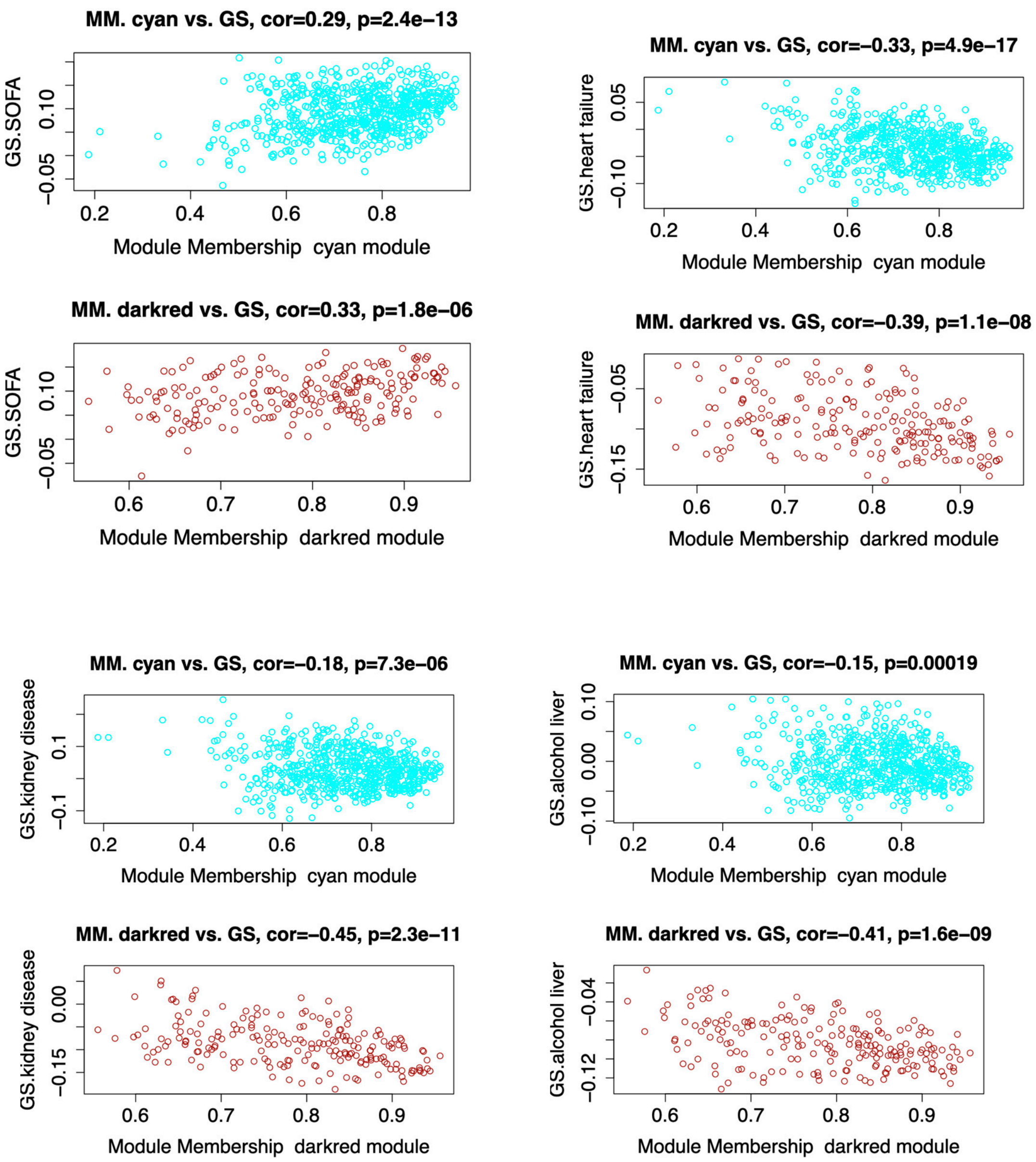

Figure S7. Scatterplots of gene significance for EOD, SOFA score, BUN, LDH, Alcohol/non-alcohol liver disease, heart failure and chronic artery disease versus module membership in MEcyan and MEdarkred modules. Gene Significance (GS) indicate how significance the gene in the modules are significant to the trait and Module Membership(MM)

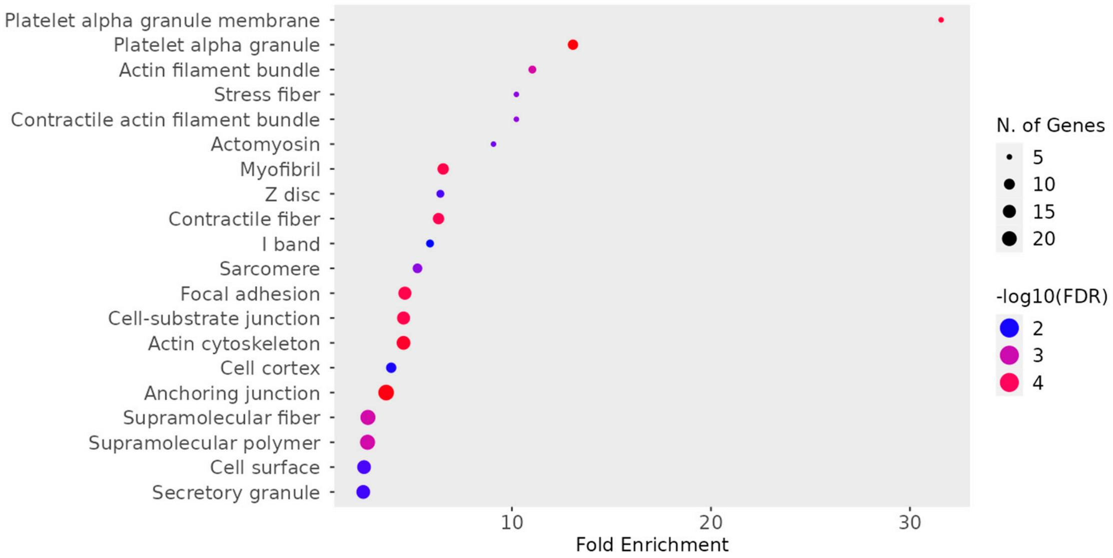

Figure S8. Dot plot for top 20 enriched cellular component base on based for gene module, MEdarkred based on GO annotation

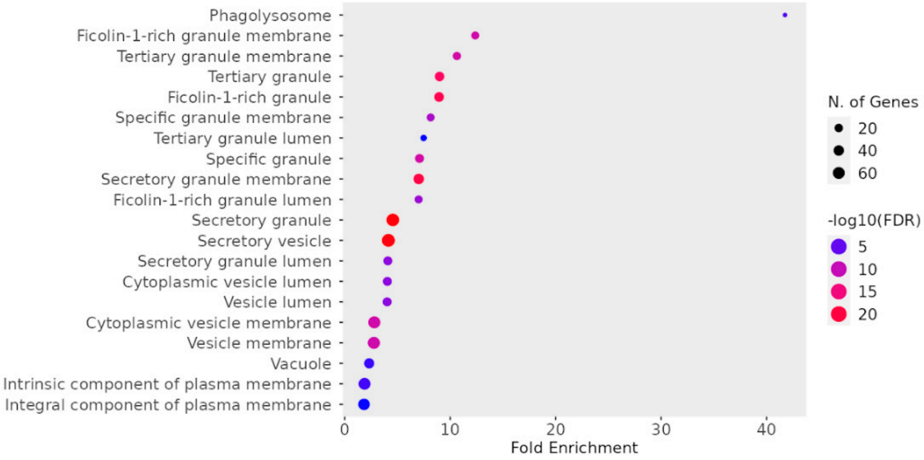

Figure S9. Dot plot for top 20 most enriched cellular components for gene module, MEcyan based on GO annotation
